# Serum Growth Differentiation Factor-15/Albumin Ratio as a 2-Year Survival Marker of End-Stage Renal Disease Patients Initiating Maintenance Hemodialysis

**DOI:** 10.3390/diagnostics12020257

**Published:** 2022-01-20

**Authors:** Eu-Jin Lee, Haet-Bit Hwang, Soo-Hyun Han, Young-Rok Ham, Jin-Ah Shin, Kang-Wook Lee, Ki-Ryang Na, Dae-Eun Choi

**Affiliations:** 1 Department of Nephrology, Chungnam National University Hospital, Daejeon 35015, Korea; eujinlee@cnuh.co.kr (E.-J.L.); hhb02@cnuh.co.kr (H.-B.H.); medihsh@cnuh.co.kr (S.-H.H.); youngrok01@cnuh.co.kr (Y.-R.H.); kwlee@cnu.ac.kr (K.-W.L.); 2 Department of Medical Science, Medical School, Chungnam National University, Daejeon 35015, Korea; wlsdkahh@o.cnu.ac.kr

**Keywords:** GDF-15, end-stage renal disease, hemodialysis, survival

## Abstract

It is important to identify risk factors related to mortality in end-stage renal disease (ESRD) patients starting renal replacement therapy. Recently, several studies proposed that growth-differentiation factor-15 (GDF-15) is a possible biomarker for the prognosis of patients on maintenance hemodialysis. Here, we investigated the predictive value of serum GDF-15/Albumin ratio on two-year mortality in ESRD patients initiating maintenance hemodialysis. The study was a single center, retrospective study on ESRD patients starting maintenance hemodialysis with a follow-up of two years. All patients completed laboratory test and bioimpedance spectroscopy prior to the initiation of the first dialysis. The patients were stratified into quartiles according to the quartiles of serum GDF-15/Albumin ratio. Among the 159 patients, the mean age was 61.78 ± 12.52 years and median survival was 20.03 ± 7.73 months. The highest GDF-15/Albumin quartile was significantly more associated with the increased risk of all-cause mortality than other quartiles (unadjusted hazard ratio (HR): 8.468, 95% CI 2.981–24.054, *p* < 0.001). Older age and a higher overhydration state were associated with GDF-15/Albumin ratio. The ROC analysis confirmed that the ability of the GDF-15/Albumin ratio to predict mortality was superior to GDF-15 or albumin alone. In conclusion, the GDF-15/Albumin ratio measured at the initial maintenance hemodialysis is an independent prognostic marker of two-year mortality in ESRD patients.

## 1. Introduction

Chronic kidney disease (CKD) is one of the many increasing global public health issues causing a high socioeconomic burden to health systems [1]. As a result, the global prevalence of CKD and end-stage renal disease (ESRD) requiring renal replacement therapy (RRT) has been on the rise over the past twenty years [2]. There are international differences in selecting the modalities for RRT, however hemodialysis is the most common option worldwide [3,4].

During the last decade, the all-cause mortality of ESRD patients on maintenance hemodialysis has gradually decreased [4] owing to improvements in dialysis technology and the treatment of comorbid conditions. However, the overall mortality rate of the patients on hemodialysis is still higher than the general population and even higher in the first few months after the initiation of dialysis [4,5,6]. The risk factors for increasing mortality in this population are cardiovascular disease [7] and non-cardiovascular comorbidities such as infection and malnutrition [8]. Also, low serum albumin [9] and volume overload [10,11] contribute to an increased risk of death and hypoalbuminemia itself can affect fluid retention in ESRD patients [12,13].

Researchers have attempted to develop biomarkers to predict mortality in ESRD patients. Serum albumin, creatinine and cholesterol were the focus in the 1990s [9] and since then, C-reactive protein (CRP) [14,15], parathyroid hormone (PTH) [16], N-terminal pro-B-type natriuretic peptide (NT-ProBNP) [17], troponin I [17], fibroblast growth factor 23 (FGF-23) [18], and many other laboratory parameters have been studied.

Recently, growth differentiation factor-15 (GDF-15), also known as macrophage inhibitory cytokine-1 (MIC-1) a stress-responsive member of the transforming growth factor-β (TGF-β) cytokine superfamily, has been studied in various chronic diseases as biomarker for disease progression and mortality. GDF-15 is weakly expressed under normal physiologic conditions and upregulated in response to oxidative stress, tissue ischemia, inflammation, and metabolic stress [19,20]. Its expression is high in the placenta [21] and prostate [22] but also increased expression is detected in heart [23], kidney [24], pancreas [25], liver [26] and colon [27].

GDF-15 is also known to be elevated in various kidney diseases and recent studies have suggested that GDF-15 is a prognostic marker of disease progression and mortality in CKD patients. In a cohort study of non-dialysis CKD patients, circulating GDF-15 levels were significantly associated with increased risk of progression of CKD [24]. Tuegel et al. (2018) showed that the non-dialysis CKD patients with higher circulating levels of GDF-15 had greater mortality [28]. In addition, several studies reported that increased levels of circulating GDF-15 are associated with both higher all-cause mortality [29] and cardiovascular mortality [30,31] in patients on maintenance hemodialysis.

Meanwhile, whether GDF-15 levels measured before starting hemodialysis can reflect the survival of ESRD patients during maintenance hemodialysis remains poorly studied. Considering that the premature mortality of ESRD patients occurs more frequently within a year after the initiation of dialysis [6], it is important to develop early markers to predict unexpected deaths and identify modifiable factors to prevent mortality. Since GDF-15 levels were reported to have inverse correlation with serum albumin levels in previous studies [32,33], it seems appropriate to take albumin status into account when interpretating GDF-15.

In this study, we investigated the prognostic value of serum GDF-15 at the initiation of hemodialysis for all-cause mortality in ESRD patients and the associations between GDF-15 and clinical variables. We analyzed the role of GDF-15 in combination with serum albumin as a newly designed index, GDF-15/albumin. We assumed that GDF-15/Albumin measured at the initiation of hemodialysis is associated with increased risk of all-cause mortality in patients starting hemodialysis.

## 2. Materials and Methods

### 2.1. Study Design

This study was a single-center retrospective study at the Chungnam National University hospital, Daejeon, Republic of Korea. We reviewed the medical records of the patients who were diagnosed with ESRD, naïve to renal replacement therapy and prescribed to start maintenance hemodialysis by our division in the outpatient-clinic, emergency room, general ward or intensive care unit from May 2014 to August 2019. Nephrologists in our hospital diagnosed ESRD based on clinical features, and laboratory tests including eGFR and ultrasonographic findings of chronic kidney disease (i.e., reduced longitudinal length, reduced cortical thickness, increased renal cortical parenchymal echogenicity, and increased marginal irregularities). Maintenance hemodialysis was defined as three hemodialysis sessions per week required for the life-long replacement of renal function. All patients were encouraged to undergo full baseline evaluation on the first day of hemodialysis before the initiation of dialysis if possible. The full baseline evaluation included a bioimpedance spectroscopy (BIS) for the assessment of body composition, routine laboratory tests which were necessary for for proper treatment. Also, full evaluation included deposition of additional blood samples for extended laboratory tests requiring written consent for the single center CKD cohort conducted in our hospital. The exclusion criteria were being under age 18 or over age 85, evidence of acute kidney injury or acute kidney disease, a hemodialysis duration less than two weeks, having baseline studies not conducted before the first hemodialysis on the date of initiation, incomplete pre-dialysis bioimpedance spectroscopy results, incomplete laboratory data, or previous history of transplantation. The protocol for this study was reviewed and approved by the Institutional Review Boards of Chungnam National University Hospital (No. 2021-05-068).

### 2.2. Assessment of Body Composition and Overhydration State

The body composition and hydration state for patients was assessed with the Body Composition Monitor (Fresenius Medical Care, Bad Homburg, Germany), a portable bioimpedance spectroscopy device. All measurements were performed by one trained nurse before the start of the first hemodialysis with the patients relaxing in supine position for at least 5 min prior to the measurement. Collected data were overhydration (OH), total body water (TBW), extracellular water (ECW), intracellular water (ICW), lean tissue index (LTI), fat tissue index (FTI), lean tissue mass (LTM), fat mass, body cell mass (BCM) and dry weight. To compare the volume state between patients, the hydration state was normalized to OH/ECW and OH/ECW > 15% was considered overhydrated [34,35].

### 2.3. Assessment of Biomarker (GDF-15)

The biospecimens and data used for further analysis on GDF-15 were provide by the Biobank of Chungnam National University Hospital, a member of the Korea Biobank Network. Blood sampling to measure the level of GDF-15 was carried out at the first access to previously formed hemodialysis vascular accesses sites (arteriovenous fistula, arteriovenous graft or central venous catheter).

Within six hours of sampling, the blood samples were centrifuged and the serums were carefully stored at −80 °C in the Human Resource Tissue Bank (Biobank) at our hospital. An experienced researcher in the lab who was not privy to the patients’ clinical information performed the measurement of the GDF-15 serum levels twice using an enzyme-linked immunosorbent assay (ELISA) (human GDF-15 Quantikine ELISA kit; R&D Systems, Minneapolis, MN, USA). In this study, the average of the duplicated values of serum GDF-15 were applied for further analysis.

### 2.4. Assessment of Clinical Parameters and Comorbidities

Information regarding the patient’s baseline characteristics and comorbidities were collected by reviewing previous medical records. Major comorbidities included coronary artery disease, heart failure, cerebrovascular disease and malignancy. All laboratory tests were performed on the blood samples within 6 h prior to the start of the first hemodialysis. The estimated glomerular filtration rate (eGFR) was calculated using the Modification of Diet in Renal Disease (MDRD) equation.

### 2.5. Outcomes

The primary outcome was all-cause mortality. Deaths were identified by reviewing hospital records on the National Health Insurance Service database. Patients were censored at the last visit or when receiving kidney transplantation during the 24-month follow-up period.

### 2.6. Statistical Analysis

Data are presented as means ± standard deviatioor median (interquartile range) for continuous variables and number with percentage for categorical variables as indicated. A *p* value of <0.05 was regarded as statistically significant and two-tailed test of the hypothesis was assumed to obtain 95% confidential intervals. To compare baseline characteristics, one-way analysis of variance (ANOVA) and Kruskal-Wallis test were used for continuous variables and Chi-square tests or Fisher’s exact test were used for categorial variables. Pearson’s correlation coefficients were calculated to investigate the correlation of the GDF-15/albumin ratio and other clinical parameters. Univariate and multivariate linear regression analysis were performed to test the association of the GDF-15/albumin ratio and clinical parameters. Also, a univariate and multivariate logistic regression analysis were conducted to determine the association between the highest quartile of GDF-15/albumin ratio and continuous variables. Kaplan-Meier analysis was performed to compare the survival time for patients classified by quartiles of GDF-15/albumin ratio, and the Log-rank test was used to compare the difference in survival times. ROC analysis was used to evaluate the predictive value of GDF-15/albumin to estimate all-cause mortality within the first 24-months after the initiation of hemodialysis. Youden’s index was used for selecting the optimal cut point for each variable. All statistical analyses were performed using SPSS version 26 (SPSS Inc., Chicago, IL, USA).

## 3. Results

### 3.1. Baseline Characteristics of the Study Population

**Figure 1:**
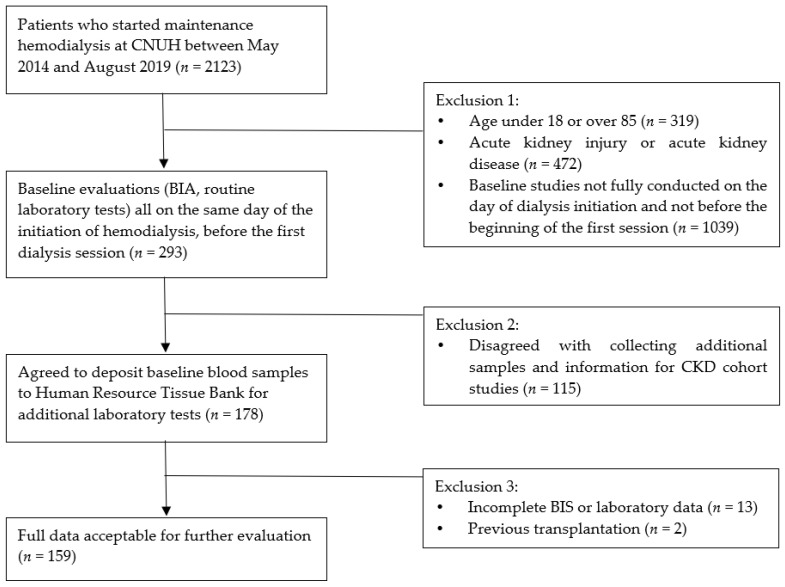
Study population.

A total of 2123 patients started maintenance hemodialysis at Chungnam National University Hospital between May 2014 and August 2019. A total of 293 patients underwent baseline evaluation including blood samples and pre-dialysis bioimpedance spectroscopy (BIS) on the same day of the initiation of hemodialysis. A total of 178 patients deposited additional blood samples for further investigation with informed consent. Finally, 159 patients were eligible for analysis and they were divided into four groups according to quartiles of GDF-15/albumin ratio (Figure 1).

The baseline characteristics of the study population are listed in Table 1. The participants had a mean age of 61.78 ± 12.52 years and mean survival period of 20.03 ± 7.73 months. The median levels of serum GDF-15 and serum albumin were 5.22 (IQR: 3.034) ng/mL and 3.50 (IQR: 0.7) g/dL, respectively.

The patients in the highest GDF-15/albumin quartile were older, more overhydrated (higher OH/ECW), and had higher levels of serum GDF-15. Compared to the other three quartiles, the patients in the highest quartile exhibited the largest numbers of mortality (*n* = 12) with the shortest mean survival (16.58 ± 7.79 months). And these patients had lower levels of total protein, albumin and albumin-to-globulin ratio (A/G ratio) with statistical significance. Furthermore, these patients were likely to have lower levels of serum blood urea nitrogen and creatinine and higher levels of eGFR although the differences among quartiles were not statistically significant. Underlying cardiovascular comorbidities did not differ across quartiles.

### 3.2. Association of GDF-15/Albumin with All-Cause Mortality

In this study population, 17 all-cause mortality events occurred during the 24-month follow-up and the causes are as follows; aspiration pneumonia (*n* = 1), septic shock (*n* = 2), multiorgan failure (*n* = 2), multiple myeloma (*n* = 1), chronic myeloid leukemia (*n* = 1), acute subdural hemorrhage (*n* = 1). The exact cause of death in remaining nine patients were impossible to assess since the mortality events occurred outside of our hospital and the information from the National Health Insurance Service database lacked the exact cause of death.

**Figure 2:**
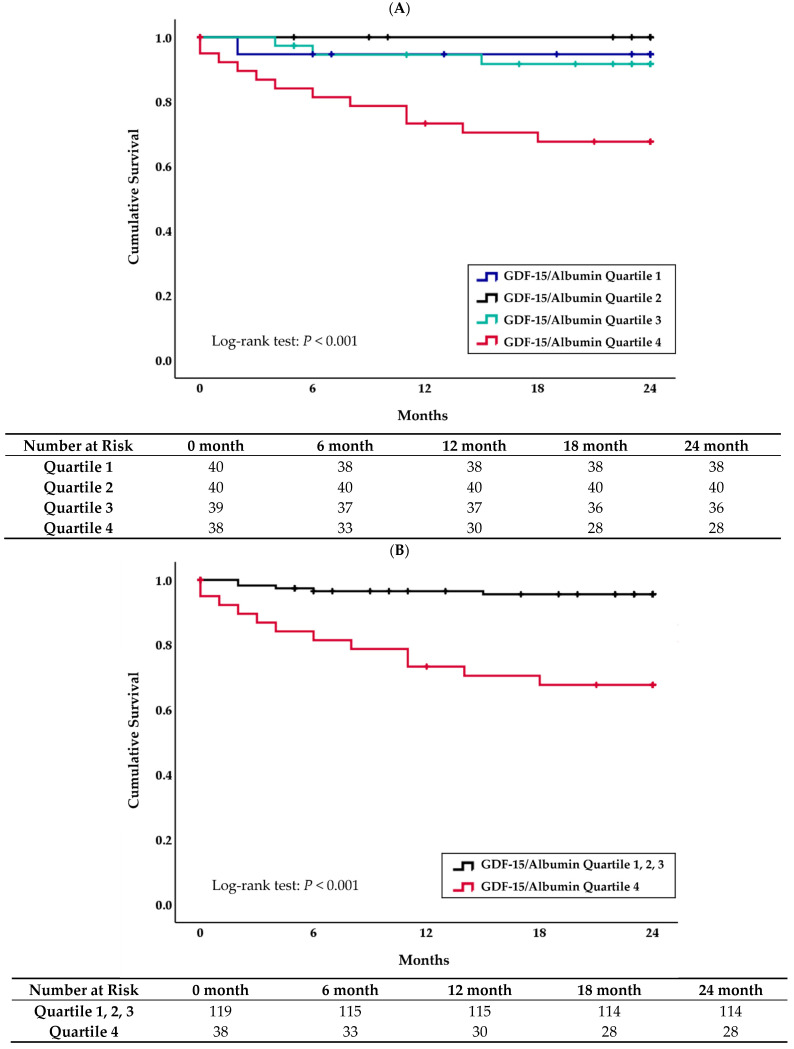
Kaplan-Meier analysis of all-cause mortality in ESRD patients starting maintenance hemodialysis according to: (**A**) each GDF-15/albumin quartile and (**B**) highest GDF-15/albumin quartile.

Kaplan-Meier survival analysis indicated that the patients in the highest GDF-15/albumin quartile were significantly associated with an increased risk of all-cause mortality (Log-rank *p* < 0.001) than the other three quartiles (Figure 2A,B).

### 3.3. Association of GDF-15/Albumin with Clinical and Biochemical Variables

Correlations with GDF-15/albumin and clinical and biochemical parameters were examined with Pearson’s correlation coefficients. Age (*r* =0.218, *p* = 0.006), OH/ECW (*r* = 0.305, *p* < 0.001), OH (*r* = 0.317, *p* < 0.001), total cholesterol (*r* = 0.181, *p =* 0.026), and eGFR-MDRD (*r* = 0.168, *p* = 0.034) levels were positively correlated with GDF-15/albumin while total protein (*r* = 0.408, *p* < 0.001) levels were negatively correlated with GDF-15/albumin.

In univariable linear regression analysis, GDF-15/albumin was independently associated with age, OH/ECW, total cholesterol, and eGFR (MDRD) (Table 2). Also, in multivariable analysis, increases in age and OH/ECW were related to higher GDF-15/Albumin with statistical significance.

### 3.4. Increased Risk of All-Cause Mortality in Patients with Higher GDF-15/Albumin

In unadjusted Cox proportional hazard analysis, the highest quartile of GDF-15/albumin level was more significantly associated with increasing risk of all-cause mortality than the other three quartiles (Hazard ratio (HR): 8.468, 95% CI 2.981–24.054, *p* < 0.001) during the first 24-months after starting maintenance hemodialysis (Table 3). After multivariable adjustments with other risk factors, the highest quartile of GDF-15/albumin level still remained as a greater risk of all-cause mortality.

### 3.5. Potential Value of GDF-15/Albumin for Prediction of All-Cause Mortality 

**Figure 3:**
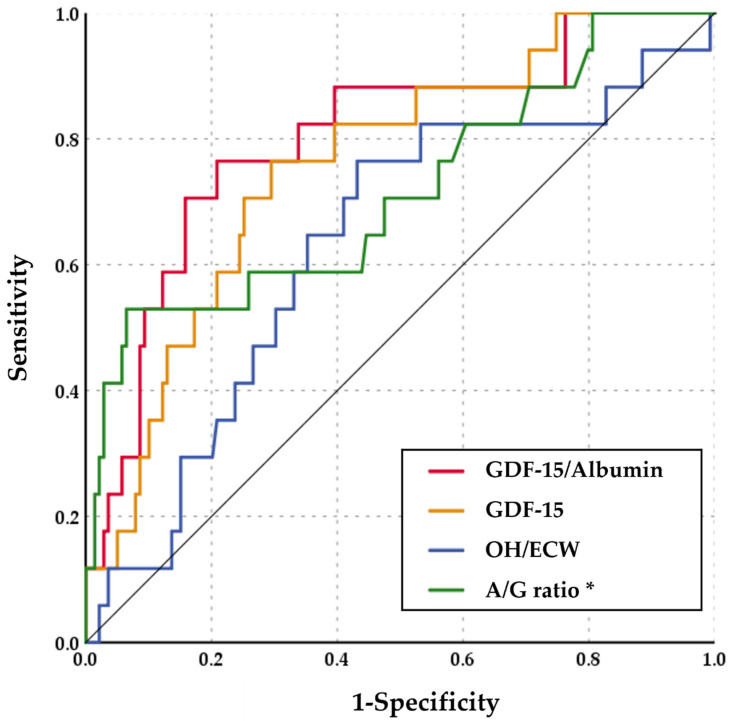
ROC analysis comparing sensitivity and specificity of GDF-15/albumin, GDF-15, OH/ECW, and A/G ratio for predicting all-cause mortality. * The test direction of ROC curve analysis of A/G ratio was opposite from that of GDF-15/Albumin, GDF-15 and OH/ECW.

We performed ROC analysis to examine the predictive accuracy of all-cause mortality of GDF-15/albumin (Figure 3). According to this analysis, the area the under the curve (AUC) for GDF-15/albumin was GDF-15/Albumin was 0.801 (95% CI: 0.685–0.917, *p* < 0.001), 0.758 for GDF-15 alone (95% CI: 0.644–0.872, *p* = 0.001), 0.715 for A/G ratio (95% CI: 0.567–0.862, *p* = 0.004) and 0.638 for OH/ECW (95% CI: 0.489–0.773, *p* = 0.078). Serum albumin had an AUC of 0.766 (95% CI: 0.623–0.908, *p* < 0.001). Overall, GDF-15/albumin was a better predictor for all-cause mortality than GDF-15 alone, the overhydration state, A/G ratio or albumin in patients starting maintenance hemodialysis. The optimal cut-off value of GDF-15/albumin was 219.23 ng/g with a sensitivity of 76.5% and a specificity of 79.1%.

## 4. Discussion

Here, we demonstrated that an elevated GDF-15/albumin ratio measured at the initiation of hemodialysis is associated with all-cause mortality within two years. The association was independent of all-cause mortality after adjustment for age, diabetes mellitus, heart failure, OH/ECW and eGFR. In addition, the predictive value of the GDF-15/albumin ratio was better than that of GDF-15 alone or other markers such as overhydration status or hypoalbuminemia proven by ROC analysis. In Pearson’s correlation coefficient analysis, GDF-15/Albumin showed positive correlations with age, OH/ECW, OH, total cholesterol and eGFR. For the multivariable linear regression models (Table 2), we selected the variables that had β with a *p*-value less than 0.05, which was considered statistically significant in univariable linear regression analysis. For model 2 in multivariable linear regression, we selected age and OH/ECW since these two markers were previously reported to have association with increased mortality in hemodialysis patients. For model 3, we included all variable with statistical significance to compare the independent power in the regression model. As a result, we confirmed that age and OH/ECW were eligible for prediction of GDF-15/Albumin.

Previous studies showed correlations between GDF-15 and various kidney disease. Studies conducted from our hospital showed that GDF-15 is a predictor of the progression of membranous nephropathy [36] and adverse outcomes in immunoglobulin A nephropathy [37]. For chronic kidney disease, one study demonstrated that circulating levels of plasma GDF-15 were strongly correlated with the intrarenal expression of GDF-15 and was markedly associated with an increased risk of CKD progression [24]. A population prospective cohort study carried out in Sweden reported that increased GDF-15 levels were related with incident CKD and a decline in eGFR in the general population, independent of potential risks of death like cardiovascular disease and diabetes [38]. Another study showed that higher circulating GDF-15 levels were correlated to greater mortality in CKD patients and was also associated with a higher risk of heart failure [28]. Some studies with mouse models showed protective effects of GDF-15 in the kidney. In mouse models of type 1 and type 2 diabetes, damages in renal tubular systems and interstitium, rather than glomerulus, were increased in GDF-15 knock-out mice [39]. This preclinical study suggested a protective role of GDF-15 on kidney against tissue injury leading to renal dysfunction.

In terms of hemodialysis, several studies have investigated the role of GDF-15 for predicting mortality. A study by Yilmaz et al. (2015) [31] revealed that increased levels of GDF-15 were related to subclinical atherosclerosis in patients on maintenance hemodialysis, which was a result of an independent association of GDF-15 on carotid intima-media thickness. You et al. (2017) [29] and Chang et al. (2021) [30] reported that higher levels of GDF-15 were strongly associated with a higher risk of death in patients on maintenance hemodialysis. The latter study developed a GDF-15-based risk to predict mortality in the study population by a combination of GDF-15 tertiles, age and hypoalbuminemia. However, these studies all measured GDF-15 from ESRD patients already on maintenance hemodialysis, which implicates that this treatment may have an impact on the increased risk of atherosclerosis and heart failure leading to a higher mortality compared to the point at the start of hemodialysis.

Our preliminary data focused on GDF-15 alone also demonstrated that a higher GDF-15 measured at the dialysis initiation was correlated to an increased risk of death during the follow-up period (Supplementary Figure S1A,B). When the study population was stratified only by the level of serum GDF-15, the highest quartile had markedly increased numbers of deaths. However, the analysis of hazard ratio of all-cause mortality showed that GDF-15 itself was not independent from albumin (Supplementary Table S1).

Albumin is a well-known marker for inflammation as well as nutrition in various chronic diseases. The two previously mentioned studies also revealed that higher GDF-15 levels were associated with hypoalbuminemia [30,31] in maintenance hemodialysis patients. The exact mechanism between GDF-15 and hypoalbuminemia remains to be elucidated, but since GDF-15 is related to tumor-induced cachexia and hypoalbuminemia reflects malnutrition in hemodialysis patients [40], there may be a possible link between these two factors to provide more specific information on the nutritional status of ESRD patients. In this context, the newly proposed index of the GDF-15/albumin ratio could be an option for the surveillance of an increased risk of mortality in ESRD patients starting hemodialysis.

Fluid overload is a well-established risk factor for increased mortality in patients on hemodialysis. The findings from our study support previous studies showing the value of volume overload assessed with whole-body BIS on predicting all-cause mortality in ESRD patients (Supplementary Figure S2). The relative hydration state (ΔHS_rel_: hydration state/extracellular water) had a high predictive value to predict all-cause mortality in patients on maintenance hemodialysis and ΔHS_rel_ > 15% was strongly related to increased death in the study population [34]. For patients starting hemodialysis, higher OH/ECW at the start of maintenance hemodialysis was associated with overall survival in ESRD patients [11]. Exposure to chronic fluid overload for one year predicted a higher risk of death across blood pressure categories [10].

Although the exact correlation between GDF-15 and OH/ECW has not been fully evaluated, serum GDF-15 was reported to be elevated in patients with heart failure [41,42]. Generally, heart failure, at some point, is known to present with signs and symptoms of volume overload. In a recent study on biomarkers for heart failure in hemodialysis patients by Claus et al. [43], GDF-15 had a weak correlation with the volume status assessed with bioimpedance analysis, but data were not statistically significant and the amount of volume overload was small. Therefore, we presumed that GDF-15 may have a possible, indirect mechanism with volume overload and heart failure in patients on hemodialysis. The exact mechanism underlying between GDF-15, GDF-15/Albumin and volume overload requires further research.

Lower BMI in dialysis patients is also associated with higher all-cause mortality while higher BMI seems to have a protective effect on survival, partially explained by the obesity paradox [44,45]. Nonetheless, the result from our study was not statistically sufficient to support the role of a lower BMI on an adverse outcome of survival in patients on dialysis (Supplementary Figure S3).

The strength of this study is the uniformity of race and the same treatment protocol performed in a single hospital despite retrospective study. In addition, according to our knowledge, this is the first study to show that GDF-15/Albumin ratio is a useful tool for predicting survival in patients starting maintenance hemodialysis. This study may provide inspiration to further survival studies using GDF-15.

Our study has several limitations to be acknowledged. First, since this was a retrospective study, some findings may have been exposed to possible confounding factors. Second, the number of subjects in this study was relatively small. This is primarily due to the fact that we were strictly allowing cases to be included only if all baseline studies were fully completed on the same day of dialysis initiation and before the start of the first session. Third, wherever GDF-15/Albumin ratio is associated with cardiovascular disease or heart failure is not fully studied. Only a limited number of patients were evaluated by a cardiologist with previous or on-going evidence of co-existing cardiac comorbidities. Specifically, it was not a routine for patients to undergo coronary angiogram or transthoracic echocardiogram prior to starting maintenance hemodialysis. Therefore, the number of patients with underlying cardiac conditions, which may have an influence on the increase in both GDF-15/Albumin ratio and risk of early mortality, may have been underestimated.

## 5. Conclusions

In conclusion, elevations in the GDF-15/Albumin ratio at the start of the initial maintenance hemodialysis are associated with a two-year mortality in ESRD patients. GDF-15/Albumin, the newly designed marker, may be helpful to assist physician to predict early mortality and provide chances to search for modifiable factors to prevent early mortality in this population. Further prospective studies are required to determine whether the GDF-15/Albumin ratio at the start of hemodialysis is valuable for predicting long-term survival during life-long renal replacement therapy.

## Figures and Tables

**Table 1:** Baseline characteristics of the study population according to quartiles of serum GDF-15/albumin ratio.

	Overall (*n* = 159)	Quartile 1 (*n* = 40)	Quartile 2 (*n* = 40)	Quartile 3 (*n* = 39)	Quartile 4 (*n* = 40)	*p* Value
Age at hemodialysis initiation (years)	61.78 ± 12.52	56.38 ± 14.34	61.20 ± 10.92	63.078 ± 12.38	66.50 ± 10.29	0.003
Male (*n*, %)	100 (62.9%)	19 (47.5%)	24 (60.0%)	27 (69.2%)	30 (75.0%)	0.061
Median survival period (months)	20.03 ± 7.73	20.38 ± 7.77	22.73 ± 4.30	20.46 ± 6.99	16.58 ± 7.79	0.004
Number of deaths (%) ^b^	17 (10.7%)	2 (5.0%)	0 (0.0%)	3 (7.7%)	12 (30.0%)	<0.001
Etiology						
DM (%)	83 (52.2%)	12 (30.0%)	21 (52.5%)	25 (64.1%)	25 (62.5%)	0.008
HTN (%)	132 (83.0%)	34 (85.0%)	32 (80.0%)	31 (79.5%)	35 (87.5%)	0.733
GN, Nephrotic syndrome (%) ^b^	10 (6.3%)	4 (10.0%)	3 (7.5%)	1 (2.6%)	2 (5.0%)	0.261
PCKD (%) ^b^	8 (5.0%)	6 (15.0%)	1 (2.5%)	1 (2.6%)	0 (0.0%)	0.013
Others (%)	27 (17.0%)	8 (20.0%)	6 (15.0%)	5 (12.8%)	8 (20.0%)	0.775
Comorbidities (%)						
Coronary artery disease (%) ^b^	18 (11.3%)	3 (7.5%)	5 (12.5%)	5 (12.8%)	5 (12.5%)	0.867
Heart failure (%) ^b^	12 (7.5%)	2 (5.0%)	4 (10.0%)	3 (7.7%)	3 (7.5%)	0.920
Cerebrovascular disease (%) ^b^	18 (11.3%)	6 (15.0%)	3 (7.5%)	3 (7.7%)	6 (15.0%)	0.584
Malignancy (%) ^b^	11 (6.9%)	1 (2.5%)	4 (10.0%)	2 (5.1%)	4 (10.0%)	0.486
Height (cm)	162.31 ± 9.33	162.02 ± 9.47	161.43 ± 10.02	162.95 ± 8.91	162.86 ± 9.13	0.871
Weight (kg)	65.23 ± 14.29	64.19 ± 13.66	66.18 ± 14.01	66.56 ± 14.23	64.03 ± 15.53	0.803
Systolic blood pressure (mmHg)	147.15 ± 22.79	140.32 ± 16.83	143.82 ± 20.36	151.82 ± 23.59	152.14 ± 27.23	0.079
Diastolic blood pressure (mmHg) ^a^	78.00 (18)	80.00 (14)	70.00 (23)	77.50 (18)	80.00 (64)	0.237
BMI (kg/m^2^) ^a^	24.20 (5.50)	23.30 (5.82)	25.10(4.85)	25.70 (5.70)	23.45 (4.82)	0.306
OH (L) ^a^	2.50 (3.9)	1.55 (3.3)	1.25 (1.9)	3.50 (4.1)	4.45 (4.2)	<0.001
TBW (L) ^a^	35.60 (13.30)	33.00 (11.88)	34.70 (14.53)	37.30 (13.60)	37.15 (13.03)	0.289
ECW (L) ^a^	17.10 (6.50)	15.70 (5.85)	16.40 (6.65)	18.50 (7.10)	18.75 (7.28)	0.025
ICW (L) ^a^	18.40 (6.9)	17.70 (5.8)	17.80 (7.9)	19.00 (5.7)	18.75 (7.5)	0.918
E/I ratio (ECW/ICW) ^a^	0.94 (0.20)	0.88 (0.178)	0.90 (0.150)	0.98 (0.210)	1.03 (0.267)	<0.001
OH/ECW (%)	15.54 ± 12.49	10.43 ± 10.06	10.30 ± 9.39	18.21 ± 11.93	23.27 ± 13.42	<0.001
LTI (kg/m^2^)	15.25 ± 3.38	15.00 ± 2.87	15.25 ± 3.69	15.46 ± 3.00	15.31 ± 3.99	0.944
FTI (kg/m^2^) ^a^	7.50 (5.9)	8.10 (6.2)	7.95 (6.3)	7.20 (5.5)	6.90 (6.3)	0.095
LTM (kg)	40.64 ± 11.50	39.73 ± 9.85	10.46 ± 12.70	41.61 ± 10.75	40.81 ± 12.76	0.911
Fat (kg) ^a^	14.60 (10.6)	14.60 (12.2)	15.40 (11.2)	12.60 (11.7)	13.40 (10.2)	0.113
BCM (kg)	23.35 ± 7.70	22.69 ± 6.50	23.24 ± 8.53	23.93 ± 7.16	23.54 ± 8.62	0.909
Dry weight (kg) ^a^	61.54 (17.50)	61.35 (19.25)	64.30 (19.20)	62.50 (14.80)	57.48 (14.17)	0.101
Hemoglobin (g/dL)	9.56 ± 1.33	9.84 ± 1.28	9.67 ± 1.22	9.53 ± 1.37	9.20 ± 1.41	0.163
Total protein (g/dL) ^a^	6.30 (1.0)	6.70 (0.7)	6.30 (1.0)	6.20 (1.1)	6.20 (1.1)	<0.001
Albumin (g/dL) ^a^	3.50 (0.7)	3.80 (0.5)	3.50 (0.7)	3.50 (0.9)	3.15 (1.0)	<0.001
A/G ratio	1.21 ± 0.29	1.38 ± 0.23	1.33 ± 0.25	1.16 ± 0.16	1.00 ± 0.29	<0.001
Total cholesterol (mg/dL) ^a^	148.0 (53)	141.0 (35)	144.5 (52)	157.0 (64)	158.0 (55)	0.116
Blood urea nitrogen (mg/dL)	89.63 ± 45.57	102.29 ± 75.69	90.68 ± 31.37	85.94 ± 27.82	79.52 ± 24.76	0.148
Creatinine (mg/dL)	8.93 ± 3.77	8.82 ± 3.43	9.09 ± 3.31	9.2 ± 4.13	8.42 ± 4.19	0.687
eGFR (mL/min/1.73m^2^) ^a^	6.30 (3.60)	6.35 (3.17)	6.05 (4.20)	6.20 (3.70)	6.65 (4.47)	0.521
Total calcium (mg/dL) ^a^	7.90 (1.3)	8.30 (1.0)	8.00 (1.2)	7.70 (1.7)	7.70 (1.2)	0.017
Corrected calcium (mg/dL) ^a^	8.40 (1.26)	8.48 (1.06)	8.27 (1.21)	8.22 (1.66)	8.58 (1.40)	0.091
Phosphate (mg/dL) ^a^	5.20 (1.92)	5.20 (1.70)	4.80 (2.80)	5.60 (1.80)	5.20 (1.88)	0.599
Potassium (mEq/L)	5.11 ± 0.88	5.13 ± 0.92	5.19 ± 0.73	5.10 ± 0.82	5.01 ± 1.05	0.842
CRP (mg/dL) ^a^	0.20 (0.70)	0.10 (0.40)	0.20 (0.60)	0.20 (0.70)	0.60 (2.3)	0.006
HbA1c (%) ^a^	5.90 (1)	5.30 (1)	5.60 (2)	6.00 (2)	6.30 (2)	0.014
Ferritin (ng/mL) ^a^	235.00 (272)	214.00 (182)	251.00 (290)	251.00 (342)	208.00 (274)	0.134
Total CO_2_ (mEq/L)	18.91 ± 4.39	18.67 ± 4.52	18.35 ± 4.87	18.54 ± 4.24	19.98 ± 3.90	0.354
PTH (pg/mL) ^a^	223.27 (218)	257.79 (277)	265.22 (307)	200.80 (155)	179.78 (241)	0.015
GDF-15/Albumin (ng/g) ^a^	156.71 (119.41)	89.23 (24.72)	133.09 (19.67)	187.01 (30.32)	318.02 (180.42)	<0.001
GDF-15 (ng/mL) ^a^	5.22 (3.034)	3.44 (1.172)	4.86 (0.632)	5.89 (1.654)	8.62 (4.668)	<0.001

Note: Data are displayed as mean ± standard deviation for continuous variables or median (interquartile range) for continuous variables and number (percent) for categorical variables. ^a^ Kruskal-Wallis test. ^b^ Fisher’s exact test. Abbreviations: GDF-15, growth differentiation factor-15; DM, diabetes mellitus; HTN, hypertension; GN, glomerulonephritis; PCKD, polycystic kidney disease; BMI, body mass index; OH, overhydration; TBW, total body water; ECW, extracellular water; ICW, intracellular water; E/I ratio, extracellular water/intracellular water ratio; OH/ECW, overhydration/extracellular water; LTI, lean tissue index; FTI, fat tissue index; BCM, body cell mass; A/G ratio, albumin-to-globulin ratio; eGFR, estimated glomerular filtration rate (calculated by MDRD equation); CRP, C-reactive protein; PTH, parathyroid hormone.

**Table 2:** Linear regression analysis of and clinical characteristics associated with serum GDF-15/albumin ratio.

Variables	Model 1		Model 2		Model 3	
	β (95% CI)	*p* Value	β (95% CI)	*p* Value	β (95% CI)	*p* Value
Age	2.527 (0.744, 4.310)	0.006	2.732 (1.038, 4.427)	0.002	2.537 (0.726, 4.349)	0.006
OH/ECW (%)	3.541 (1.796, 5.286)	<0.001	3.694 (1.994, 5.393)	<0.001	3.023 (1.165, 4.881)	0.002
BMI	−1.262 (−6.954, 4.430)	0.662				
LTI	−3.462 (−10.207, 3.283)	0.312				
FTI	−1.254 (−6.549, 4.041)	0.640				
Total cholesterol	0.601 (0.071, 1.131)	0.026			0.252 (−0.276, 0.780)	0.347
Creatinine	−5.528 (−11.540, 0.484)	0.071				
eGFR	3.777 (0.280, 7.273)	0.034			1.755 (−1.806, 5.315)	0.332
CRP	6.319 (−0.762, 13.400)	0.080				
HbA1c	2.443 (−3.015, 7.900)	0.377				

Model 1, univariable analysis; Model 2, multivariable analysis adjusted with age and OH/ECW; Model 3, multivariable analysis adjusted with age, OH/ECW, total cholesterol, and eGFR.

**Table 3:** Association of GDF-15/albumin ratio and all-cause mortality by Cox proportional hazard analysis.

Variables	Reference (GDF-15/Albumin Quartile 1, 2, 3)		GDF-15/Albumin Quartile 4 (>75 Percentile)	
	HR (95% CI)	*p* Value	HR (95% CI)	*p* Value
Model 1	1	N/A	8.468 (2.981, 24.054)	<0.001
Model 2	1	N/A	5.507 (1.774, 17.096)	0.003
Model 3	1	N/A	5.510 (1.774, 17.144)	0.003

Model 1, unadjusted; Model 2, adjusted for age; diabetes mellitus, OH/ECW; Model 3, adjusted for age, diabetes mellitus, OH/ECW, BMI. N/A, not applicable.

## Supplementary Materials

The following supporting information can be downloaded at: https://www.mdpi.com/article/10.3390/diagnostics12020257/s1. Figure S1: Kaplan-Meier analysis of all-cause mortality in ESRD patients starting maintenance hemodialysis according to: (A) each serum GDF-15 quartile and (B) highest GDF-15 quartile; Figure S2: Kaplan-Meier analysis of all-cause mortality in ESRD patients starting maintenance hemodialysis according to OH/ECW, the normalized hydration state (OH/ECW > 15% was considered overhydrated); Figure S3: Kaplan-Meier analysis of all-cause mortality in ESRD patients starting maintenance hemodialysis according to BMI; Table S1: Association of serum GDF-15 and all-cause mortality by Cox proportional hazard analysis.

## References

[1] Hill N.R., Fatoba S.T., Oke J.L., Hirst J.A., O’Callaghan C.A., Lasserson D.S., Hobbs F.D. (2016). Global Prevalence of Chronic Kidney Disease—A Systematic Review and Meta-Analysis. PLoS ONE.

[2] Bikbov B., Purcell C.A., Levey A.S., Smith M., Abdoli A., Abebe M., Owolabi M.O. (2020). Global, regional, and national burden of chronic kidney disease, 1990–2017: A systematic analysis for the Global Burden of Disease Study 2017. Lancet.

[3] Robinson B.M., Akizawa T., Jager K.J., Kerr P.G., Saran R., Pisoni R.L. (2016). Factors affecting outcomes in patients reaching end-stage kidney disease worldwide: Differences in access to renal replacement therapy, modality use, and haemodialysis practices. Lancet.

[4] United States Renal Data System (2020). 2020 USRDS Annual Data Report: Epidemiology of Kidney Disease in the United States.

[5] Nordio M., Limido A., Maggiore U., Nichelatti M., Postorino M., Quintaliani G. (2012). Survival in patients treated by long-term dialysis compared with the general population. Am. J. Kidney Dis..

[6] Robinson B.M., Zhang J., Morgenstern H., Bradbury B.D., Ng L.J., McCullough K.P., Gillespie B.W., Hakim R., Rayner H., Fort J. (2014). Worldwide, mortality risk is high soon after initiation of hemodialysis. Kidney Int..

[7] Lindner A., Charra B., Sherrard D.J., Scribner B.H. (1974). Accelerated atherosclerosis in prolonged maintenance hemodialysis. N. Engl. J. Med..

[8] de Jager D.J., Grootendorst D.C., Jager K.J., van Dijk P.C., Tomas L.M., Ansell D., Collart F., Finne P., Heaf J.G., De Meester J. (2009). Cardiovascular and noncardiovascular mortality among patients starting dialysis. JAMA.

[9] Goldwasser P., Mittman N., Antignani A., Burrell D., Michel M.A., Collier J., Avram M.M. (1993). Predictors of mortality in hemodialysis patients. J. Am. Soc. Nephrol..

[10] Zoccali C., Moissl U., Chazot C., Mallamaci F., Tripepi G., Arkossy O., Wabel P., Stuard S. (2017). Chronic Fluid Overload and Mortality in ESRD. J. Am. Soc. Nephrol..

[11] Kim Y.J., Jeon H.J., Kim Y.H., Jeon J., Ham Y.R., Chung S., Choi D.E., Na K.R., Lee K.W. (2015). Overhydration measured by bioimpedance analysis and the survival of patients on maintenance hemodialysis: A single-center study. Kidney Res. Clin. Pract..

[12] Hung S.C., Kuo K.L., Peng C.H., Wu C.H., Lien Y.C., Wang Y.C., Tarng D.C. (2014). Volume overload correlates with cardiovascular risk factors in patients with chronic kidney disease. Kidney Int..

[13] Cigarran S., Barril G., Cirugeda A., Bernis C., Aguilera A., Sanz P., Herraez I., Alegre L., Selgas R. (2007). Hypoalbuminemia is also a marker of fluid excess determined by bioelectrical impedance parameters in dialysis patients. Ther. Apher Dial..

[14] Korevaar J.C., van Manen J.G., Dekker F.W., de Waart D.R., Boeschoten E.W., Krediet R.T. (2004). Effect of an increase in C-reactive protein level during a hemodialysis session on mortality. J. Am. Soc. Nephrol..

[15] Bazeley J., Bieber B., Li Y., Morgenstern H., de Sequera P., Combe C., Yamamoto H., Gallagher M., Port F.K., Robinson B.M. (2011). C-reactive protein and prediction of 1-year mortality in prevalent hemodialysis patients. Clin. J. Am. Soc. Nephrol..

[16] Tentori F., Blayney M.J., Albert J.M., Gillespie B.W., Kerr P.G., Bommer J., Young E.W., Akizawa T., Akiba T., Pisoni R.L. (2008). Mortality risk for dialysis patients with different levels of serum calcium, phosphorus, and PTH: The Dialysis Outcomes and Practice Patterns Study (DOPPS). Am. J. Kidney Dis..

[17] Madsen L.H., Ladefoged S., Corell P., Schou M., Hildebrandt P.R., Atar D. (2007). N-terminal pro brain natriuretic peptide predicts mortality in patients with end-stage renal disease in hemodialysis. Kidney Int..

[18] Gutiérrez O.M., Mannstadt M., Isakova T., Rauh-Hain J.A., Tamez H., Shah A., Smith K., Lee H., Thadhani R., Jüppner H. (2008). Fibroblast growth factor 23 and mortality among patients undergoing hemodialysis. N. Engl. J. Med..

[19] Breit S.N., Johnen H., Cook A.D., Tsai V.W., Mohammad M.G., Kuffner T., Zhang H.P., Marquis C.P., Jiang L., Lockwood G. (2011). The TGF-β superfamily cytokine, MIC-1/GDF15: A pleotrophic cytokine with roles in inflammation, cancer and metabolism. Growth Factors.

[20] Wiklund F.E., Bennet A.M., Magnusson P.K., Eriksson U.K., Lindmark F., Wu L., Yaghoutyfam N., Marquis C.P., Stattin P., Pedersen N.L. (2010). Macrophage inhibitory cytokine-1 (MIC-1/GDF15): A new marker of all-cause mortality. Aging Cell.

[21] Sugulle M., Dechend R., Herse F., Weedon-Fekjaer M.S., Johnsen G.M., Brosnihan K.B., Anton L., Luft F.C., Wollert K.C., Kempf T. (2009). Circulating and placental growth-differentiation factor 15 in preeclampsia and in pregnancy complicated by diabetes mellitus. Hypertension.

[22] Brown D.A., Lindmark F., Stattin P., Bälter K., Adami H.O., Zheng S.L., Xu J., Isaacs W.B., Grönberg H., Breit S.N. (2009). Macrophage inhibitory cytokine 1: A new prognostic marker in prostate cancer. Clin. Cancer Res..

[23] Xu J., Kimball T.R., Lorenz J.N., Brown D.A., Bauskin A.R., Klevitsky R., Hewett T.E., Breit S.N., Molkentin J.D. (2006). GDF15/MIC-1 functions as a protective and antihypertrophic factor released from the myocardium in association with SMAD protein activation. Circ. Res..

[24] Nair V., Robinson-Cohen C., Smith M.R., Bellovich K.A., Bhat Z.Y., Bobadilla M., Brosius F., de Boer I.H., Essioux L., Formentini I. (2017). Growth Differentiation Factor-15 and Risk of CKD Progression. J. Am. Soc. Nephrol..

[25] Koopmann J., Buckhaults P., Brown D.A., Zahurak M.L., Sato N., Fukushima N., Sokoll L.J., Chan D.W., Yeo C.J., Hruban R.H. (2004). Serum macrophage inhibitory cytokine 1 as a marker of pancreatic and other periampullary cancers. Clin. Cancer Res..

[26] Liu X., Chi X., Gong Q., Gao L., Niu Y., Chi X., Cheng M., Si Y., Wang M., Zhong J. (2015). Association of serum level of growth differentiation factor 15 with liver cirrhosis and hepatocellular carcinoma. PLoS ONE.

[27] Wallin U., Glimelius B., Jirström K., Darmanis S., Nong R.Y., Pontén F., Johansson C., Påhlman L., Birgisson H. (2011). Growth differentiation factor 15: A prognostic marker for recurrence in colorectal cancer. Br. J. Cancer.

[28] Tuegel C., Katz R., Alam M., Bhat Z., Bellovich K., de Boer I., Brosius F., Gadegbeku C., Gipson D., Hawkins J. (2018). GDF-15, Galectin 3, Soluble ST2, and Risk of Mortality and Cardiovascular Events in CKD. Am. J. Kidney Dis..

[29] You A.S., Kalantar-Zadeh K., Lerner L., Nakata T., Lopez N., Lou L., Veliz M., Soohoo M., Jing J., Zaldivar F. (2017). Association of Growth Differentiation Factor 15 with Mortality in a Prospective Hemodialysis Cohort. Cardiorenal Med..

[30] Chang J.F., Chen P.C., Hsieh C.Y., Liou J.C. (2021). A Growth Differentiation Factor 15-Based Risk Score Model to Predict Mortality in Hemodialysis Patients. Diagnostics.

[31] Yilmaz H., Çelik H.T., Gurel O.M., Bilgic M.A., Namuslu M., Bozkurt H., Ayyildiz A., Inan O., Bavbek N., Akcay A. (2015). Increased serum levels of GDF-15 associated with mortality and subclinical atherosclerosis in patients on maintenance hemodialysis. Herz.

[32] Breit S.N., Carrero J.J., Tsai V.W., Yagoutifam N., Luo W., Kuffner T., Bauskin A.R., Wu L., Jiang L., Barany P. (2012). Macrophage inhibitory cytokine-1 (MIC-1/GDF15) and mortality in end-stage renal disease. Nephrol. Dial. Transplant..

[33] Tarkun P., Birtas Atesoglu E., Mehtap O., Musul M.M., Hacihanefioglu A. (2014). Serum growth differentiation factor 15 levels in newly diagnosed multiple myeloma patients. Acta Haematol..

[34] Chazot C., Wabel P., Chamney P., Moissl U., Wieskotten S., Wizemann V. (2012). Importance of normohydration for the long-term survival of haemodialysis patients. Nephrol. Dial. Transplant..

[35] Wabel P., Moissl U., Chamney P., Jirka T., Machek P., Ponce P., Taborsky P., Tetta C., Velasco N., Vlasak J. (2008). Towards improved cardiovascular management: The necessity of combining blood pressure and fluid overload. Nephrol. Dial. Transplant..

[36] Ham Y.R., Song C.H., Bae H.J., Jeong J.Y., Yeo M.K., Choi D.E., Na K.R., Lee K.W. (2018). Growth Differentiation Factor-15 as a Predictor of Idiopathic Membranous Nephropathy Progression: A Retrospective Study. Dis. Markers.

[37] Na K.R., Kim Y.H., Chung H.K., Yeo M.K., Ham Y.R., Jeong J.Y., Kim K.S., Lee K.W., Choi D.E. (2017). Growth differentiation factor 15 as a predictor of adverse renal outcomes in patients with immunoglobulin A nephropathy. Intern. Med. J..

[38] Bao X., Xu B., Borné Y., Orho-Melander M., Melander O., Nilsson J., Christensson A., Engström G. (2021). Growth differentiation factor-15 and incident chronic kidney disease: A population-based cohort study. BMC Nephrol..

[39] Mazagova M., Buikema H., van Buiten A., Duin M., Goris M., Sandovici M., Henning R.H., Deelman L.E. (2013). Genetic deletion of growth differentiation factor 15 augments renal damage in both type 1 and type 2 models of diabetes. Am. J. Physiol. Renal Physiol..

[40] Fouque D., Kalantar-Zadeh K., Kopple J., Cano N., Chauveau P., Cuppari L., Franch H., Guarnieri G., Ikizler T.A., Kaysen G. (2008). A proposed nomenclature and diagnostic criteria for protein-energy wasting in acute and chronic kidney disease. Kidney Int..

[41] Chan M.M., Santhanakrishnan R., Chong J.P., Chen Z., Tai B.C., Liew O.W., Ng T.P., Ling L.H., Sim D., Leong K.T. (2016). Growth differentiation factor 15 in heart failure with preserved vs. reduced ejection fraction. Eur. J. Heart Fail.

[42] George M., Jena A., Srivatsan V., Muthukumar R., Dhandapani V.E. (2016). GDF 15—A Novel Biomarker in the Offing for Heart Failure. Curr. Cardiol. Rev..

[43] Claus R., Berliner D., Bavendiek U., Vodovar N., Lichtinghagen R., David S., Patecki M., Launay J.M., Bauersachs J., Haller H. (2020). Soluble neprilysin, NT-proBNP, and growth differentiation factor-15 as biomarkers for heart failure in dialysis patients (SONGBIRD). Clin. Res. Cardiol..

[44] Kalantar-Zadeh K., Streja E., Kovesdy C.P., Oreopoulos A., Noori N., Jing J., Nissenson A.R., Krishnan M., Kopple J.D., Mehrotra R. (2010). The obesity paradox and mortality associated with surrogates of body size and muscle mass in patients receiving hemodialysis. Mayo Clinic Proceedings.

[45] Agarwal R. (2011). Body mass index-mortality paradox in hemodialysis: Can it be explained by blood pressure?. Hypertension.

