# Supplementary material for: Serum Growth Differentiation Factor-15/Albumin Ratio as a 2-Year Survival Marker of End-Stage Renal Disease Patients Initiating Maintenance Hemodialysis
Source: Diagnostics (Basel). 2022 Jan 20;12(2):257. doi: 10.3390/diagnostics12020257 (PMC8870838; doi:10.3390/diagnostics12020257)
Supplement: Supplementary file 1 [file diagnostics-12-00257-s001.zip › diagnostics-1542077-supplementary.pdf]

Supplementary Data

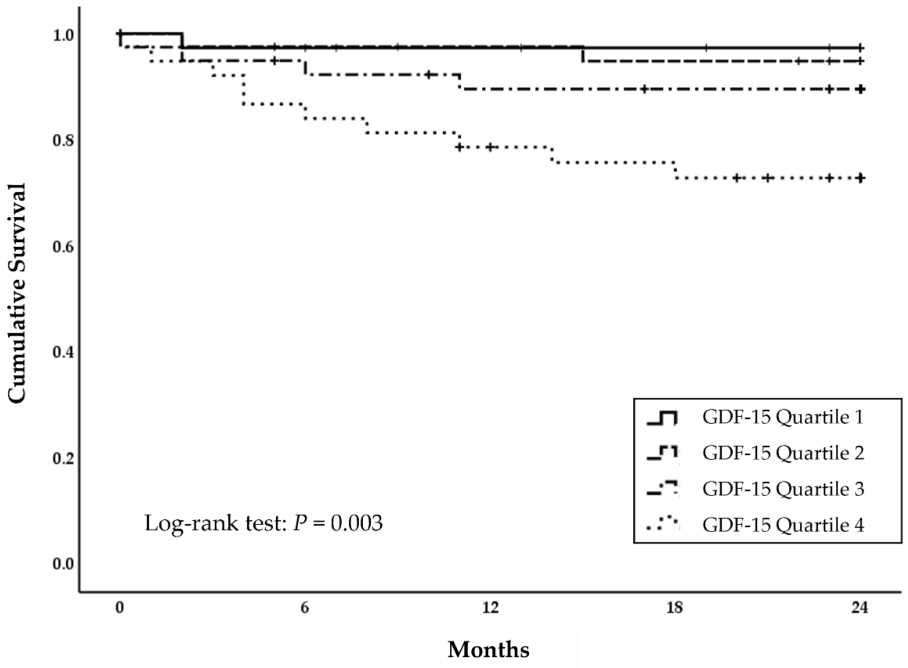

(**A**)

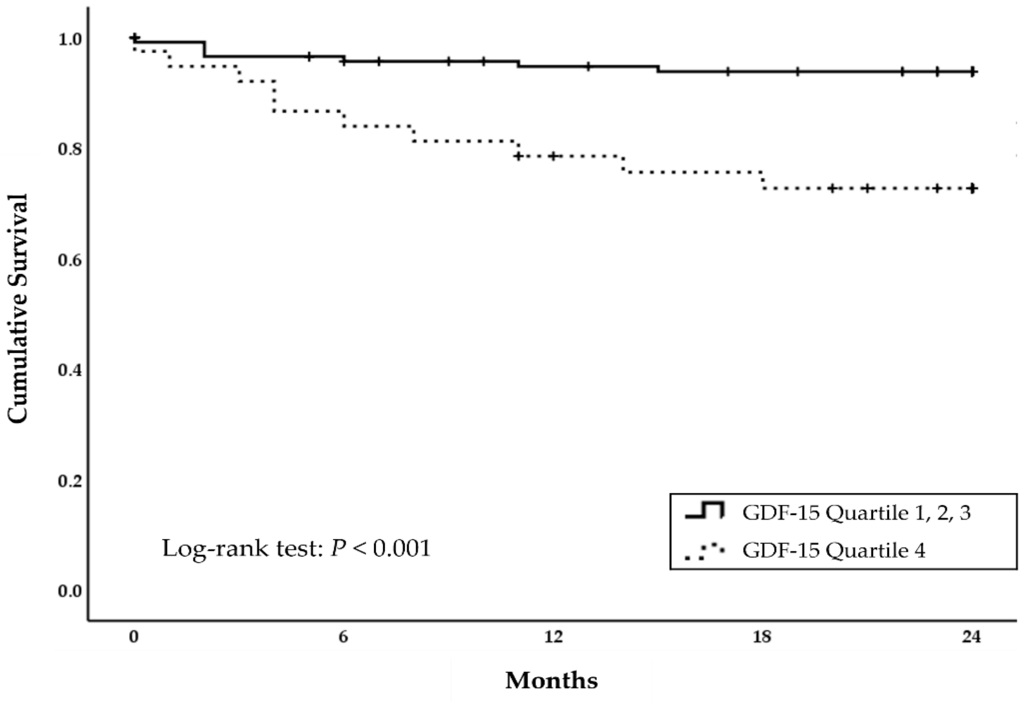

(**B**)

Figure S1. Kaplan-Meier analysis of all-cause mortality in ESRD patients starting maintenance hemodialysis according to: (**A**) each serum GDF-15 quartile and (**B**) highest GDF-15 quartile.

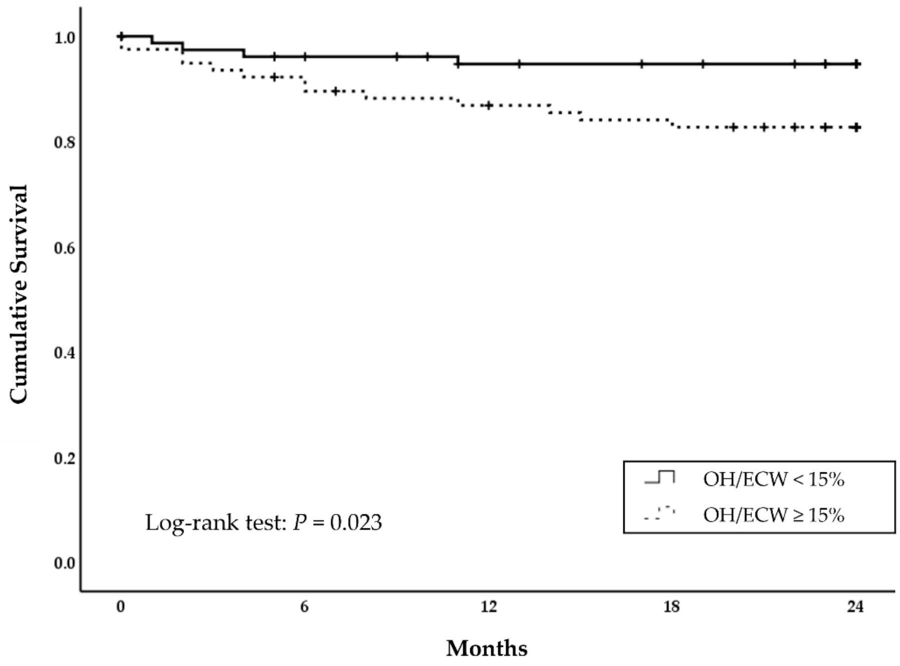

Figure S2. Kaplan-Meier analysis of all-cause mortality in ESRD patients starting maintenance hemodialysis according to OH/ECW, the normalized hydration state (OH/ECW>15% was considered overhydrated).

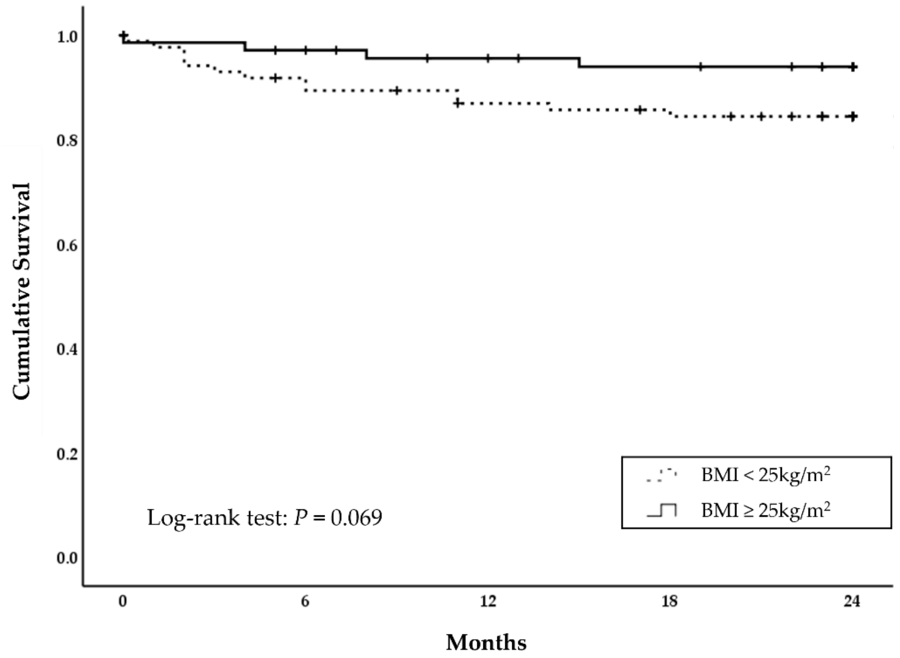

Figure S3. Kaplan-Meier analysis of all-cause mortality in ESRD patients starting maintenance hemodialysis according to BMI.

Table S1. Association of serum GDF-15 and all-cause mortality by Cox proportional hazard analysis.

Variables	GDF-15 Quartile 1, 2, 3		GDF-15/Albumin Quartile 4 (>75 percentile)	
	HR (95% CI)	P value	HR (95% CI)	P value
**Model 1**	1	N/A	4.821 (1.834, 12.673)	<0.001
**Model 2**	1	N/A	2.916 (1.022, 8.321)	0.045
**Model 3**	1	N/A	1.741 (0.614, 4.940)	0.297

Model 1: Unadjusted; Model 2: Adjusted for age, diabetes mellitus, OH/ECW, Model 3: Adjusted for age, diabetes mellitus, OH/ECW, albumin; N/A: not applicable.
